# The impact of cardiorespiratory fitness and physical workload on disability pension–a cohort study of Swedish men

**DOI:** 10.1007/s00420-023-02023-1

**Published:** 2023-11-16

**Authors:** Karin Berglund, Melody Almroth, Daniel Falkstedt, Tomas Hemmingsson, Katarina Kjellberg

**Affiliations:** 1 Unit of Occupational Medicine, Institute of Environmental Medicine, Karolinska Institutet, Solnavägen 4, 113 65 Stockholm, Sweden; 2 Center for Occupational and Environmental Medicine, Region Stockholm, Stockholm, Sweden; 3 Department of Public Health Sciences, Stockholm University, Stockholm, Sweden

**Keywords:** Ergonomics, Job-exposure matrix, Physical capacity, Musculoskeletal disorders, Cardiovascular disease, Epidemiology

## Abstract

**Objective:**

Understanding the impact of physical capacity in combination with high physical workload could be beneficial for the prevention of health-related exits from work. Therefore, the aim of this study was to investigate the separate and combined effects of low cardiorespiratory fitness and high physical workload on disability pension (DP) due to any cause, musculoskeletal disorders (MSD), and cardiovascular diseases (CVD).

**Methods:**

A total of 279 353 men born between 1951 and 1961 were followed regarding DP between 2006 and 2020, ages 45–64. Cardiorespiratory fitness was assessed during military conscription, using an ergometer bicycle test. Physical workload was based on a job-exposure matrix (JEM) linked to occupational title in 2005. Cox regression models estimated separate and combined associations with DP.

**Results:**

Low cardiorespiratory fitness and high physical workload were associated with increased risk of DP. For all cause DP, the fully adjusted hazard ratio and 95% confidence interval for those with low cardiorespiratory fitness was 1.38 (1.32–1.46) and for those with high physical workload 1.48 (1.39–1.57). For all cause and MSD DP, but not for CVD DP, the combination of low cardiorespiratory fitness and high physical workload resulted in higher risks than when adding the effect of the single exposures.

**Conclusion:**

Both low cardiorespiratory fitness in youth and later exposure to high physical workload were associated with an increased risk of DP, where workers with the combination of both low cardiorespiratory fitness and a high physical workload had the highest risks (all-cause and MSD DP).

## Introduction

Workers exposed to high physical workload have an increased risk of early exclusion from the labour market due to long-term sickness absence or disability pension (DP) (Emberland et al. 2017; Ervasti et al. 2019; Falkstedt et al. 2021; Halonen et al. 2020; Kjellberg et al. 2016). The relative physiological load by a given work task will also be higher for a worker with low physical capacity compared to a worker with high physical capacity (Krause 2010; Krause et al. 2007). Therefore, the combination of high physical workload and low physical capacity could potentially lead to an excessive physiological load and increased risks of health-related exits from work. For this reason, a better understanding of the impact of physical capacity in combination with high physical workload, could benefit preventative measures to reduce health-related exits from work. However, only a few prospective studies have explored the combination of physical capacity and physical workload on health and health-related exit from work.

A Danish cohort study found associations between high physical workload and all-cause and cardiovascular (CVD) mortality among men with poor and moderate cardiovascular fitness, but not among men with high fitness (Holtermann et al. 2010). A later Danish cohort (Holtermann et al. 2016) showed an increased risk of CVD mortality for those with high compared to low physical workload, and for those with low compared to high cardiorespiratory fitness. They also demonstrated an increased risk of CVD mortality due to high physical workload, among those with low fitness, but not among those with moderate or high fitness after adjustment for life-style factors. Furthermore, those with the combination of high physical workload and low cardiorespiratory fitness had an increased risk of CVD mortality compared to those unexposed to both factors. A Dutch prospective study investigated if a potential imbalance between muscular strength and exposure to high physical workload were associated with increased risk of neck-or back pain three years later (Hamberg-van Reenen et al. 2006). They found that combined low muscle strength and high physical workload did not entail a higher risk of neck/back disorders than exposure to high physical workload only, or low muscle strength only. Only one longitudinal study investigating effects of combined exposures to high physical workload and low cardiorespiratory fitness on DP has been found (Karpansalo et al. 2002). This study reported that high physical workload was associated with an increased risk of DP, with the strongest association for those with low cardiorespiratory fitness compared to those with high fitness.

One limitation with the aforementioned studies is the use of self-reported measurements on physical workload, except for the Dutch study that for some of the workers added assessments of workload from video recordings at workplaces to the self-reports (Hamberg-van Reenen et al. 2006). The use of self-reported data on physical workload could potentially lead to biased estimates, where participants with pre-existing health conditions at baseline may overestimate their exposure to physical workload (Gupta et al. 2018). Another approach to measure exposure to physical workload is the use of a Job Exposure Matrix (JEM). A JEM is based on aggregated exposure measurements on an occupational level which is linked to the participants based on their occupation, instead of using self-reported exposure measurement from the individuals included in the study.

Furthermore, previous studies have had limited opportunities to control for confounding factors from before labour market entry, which could be a potential problem when studying the association between physical fitness, work, and health-related outcomes (Amick et al. 2016). For instance, lower cognitive ability has been found to be correlated with lower cardiorespiratory fitness in youth (Åberg et al. 2009), as well as later DP (Lie et al. 2017; Sörberg et al. 2013). Low socioeconomic position in childhood has also been found to be associated with later DP (Krokstad et al. 2002), even when controlling for high physical workload (Månsson et al. 1998). Therefore, some of the DP cases among those with exposure to high physical workload could, to some extent, also be a result of selection forces from before labour market entry, into education and occupation.

The aim of this study is to investigate the separate and combined effects of low cardiorespiratory fitness in youth and later high physical workload and DP, due to any cause, MSD, and CVD, respectively. Our hypothesis is that low cardiorespiratory fitness aggravates the risk of DP due to high physical workload. This is investigated using a JEM and with the possibility to include potentially important confounding factors from before labour market entry.

## Methods

### Study population and data collection

**Fig. 1:**
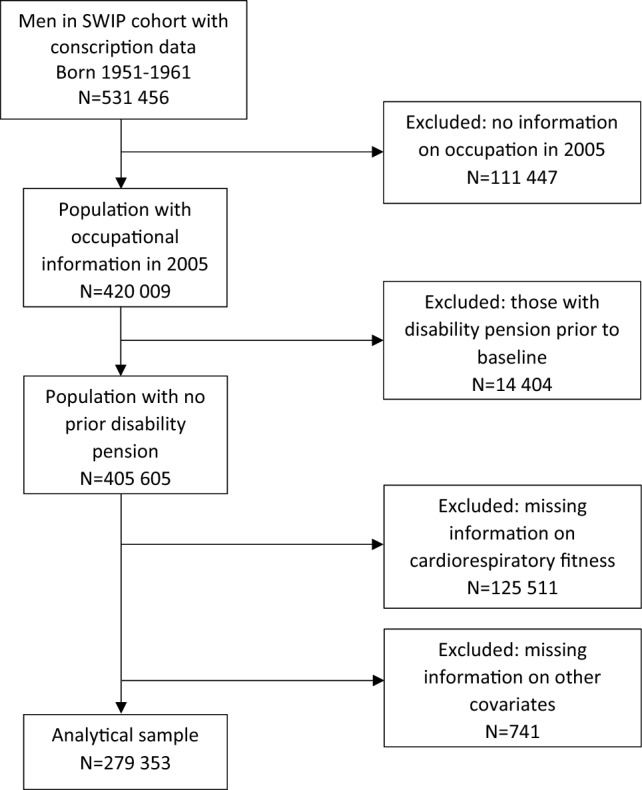
Selection of study population

The present study uses a register-based cohort consisting of linkages between administrative, medical, and social insurance registers in Sweden. Data is obtained from the Swedish Work, Illness, and labor-market Participation (SWIP) cohort, which consist of all individuals between the age of 16 and 64 years old, registered as living in Sweden in 2005 (Falkstedt et al. 2021). We restricted the study to men born between 1951 and 1961, who took part in the military conscription during the years 1969–1979, which include 531 456 individuals. At that time, conscription for military service was mandatory in Sweden and included approximately 90% of all men at the age of 18–20. The conscription consisted of two days of medical, physical, and psychological examinations (Ludvigsson et al. 2022). Of the men born 1951 to 1953, cardiorespiratory fitness was only recorded for those who conscripted after 1972. Therefore, all men who were missing information on cardiorespiratory fitness were excluded. Participants were also excluded if there were missing data on occupation in 2005, or if they had a disability pension (part- or full-time) prior to baseline (before the age of 44–54). A total of 279 353 men met all of these criteria, making this the analytical sample (Fig. 1).

### Exposures

*Cardiorespiratory fitness* was obtained from the Swedish Military Service Conscription Register and measured with an ergometer bicycle test (Wmax test). After a normal electrocardiogram (ECG), the participants performed a maximal cycle test where it is required to cycle until reaching exhaustion. Initial resistance was determined by body weight and after 5 min of bicycling reaching a pulse between 120 and 170 beats/min, resistance was increased by 25 W per minute until the conscript reached exhaustion (Ludvigsson et al. 2022). Results were recorded in watts (W). The Wmax test has shown a strong correlation with direct measurement of maximal oxygen uptake (VO _2_ max) (Andersen 1995). In the present study, the results of the bicycle test were categorized into tertiles as having “low” (<228 W), “medium” (229–255 W) or “high” (> 256 W) cardiorespiratory fitness.

*Physical workload* was classified using a Swedish JEM, previously described by Falkstedt et al. (2021) and Badarin et al. (2021). In summary, the JEM is based on responses to eight questions on physical workload, from the Swedish Work Environment Surveys between 1997 and 2013. The JEM provides sex-specific mean values for 355 different occupations, coded with the Swedish Standard Classification of Occupation (SSYK) 96 system. In this study, the level of overall physical workload is estimated based on the mean JEM values for men, for five different physical exposures. These questions were chosen to represent an overall physical workload, both mechanical load on the musculoskeletal system and strenuous work with loads on the cardiovascular system. The included exposures were heavy lifting (≥ 15 kg), physically strenuous work, working in a forward bent position, working in a twisted posture, and working with hands above shoulders. An index mean-value was computed by summing the scores for these questions and calculating the mean value. The mean values were linked to the participants through their occupational code in 2005. Data on the participants occupation were obtained from the Longitudinal Integration Database for Health Insurance and Labour Market Studies (LISA) (Ludvigsson et al. 2019) for the baseline in 2005 when the participants were between the ages of 44 and 54. The index mean value was categorized into tertiles (*“low”*, *“medium”* or *“high”* physical workload) based on the total population with complete information on occupation.

### Outcome

*Disability pension* (DP)*,* in Sweden from the year 2003 termed ‘sjukersättning’ (i.e. sickness compensation), is an applicable compensation for those between the ages 19 and 64 with an illness or disability, who are unlikely to be able to work full time in any type of work in the labour market (Forsakringskassan.se 2022). Information on DP is registered yearly in the Swedish Social Insurance Agency’s register, which include information on granting date and the associated diagnosis. In this study, cases of DP (part- or full-time) were collected between 2006 and 2020, when the participants were between the age of 45 and 55 at the start of follow up and between the age of 55 and 64 at the end of follow up. The diagnoses associated with the DP were registered according to the International Classification of Diseases (ICD 10). Cases were classified as having a DP due to any cause, and specifically to either musculoskeletal (ICD 10 codes M00-M99) or cardiovascular (ICD 10 codes I00-I99) diseases.

### Covariates

*Cognitive ability* was assessed during conscription using four different tests which were added together into scores and categorized according to their stanine distribution. In the present study, this was further categorized to indicate low (1–4), medium (5–7), or high (8–9) cognitive ability.

*Body Mass Index (BMI)* was calculated based on the height and weight reported during conscription (kg/m2) and was categorized as underweight (< 18.5), normal (18.5–24.9), overweight (25–29.9), or obese (≥ 30).

*Socioeconomic position (SEP)* during childhood was measured based on information on parents’ occupation, classified as unskilled manual workers, skilled manual workers, assistant non-manual workers, intermediate non-manual workers, high level non-manual workers, farmers, and those with no occupation reported. Parents occupational information was taken from the 1970 census when index persons were between the age of 9 and 19. Father’s occupation was the primary source of information, but mother’s occupation was used when this information was not available.

*Level of education* was measured in 2005 and obtained from the LISA register (Ludvigsson et al. 2019). It was categorized according to the number of years of education and grouped as less than or equal to nine years (compulsory school), ten to eleven years (some upper secondary), twelve years (upper secondary), or 13 or more years (university).

*Previous unemployment* was measured during the years 2001 to 2005 and was categorized as none, 1–365 days, and more than 365 days.

### Statistical analysis

Cox regression models with age as the underlying time scale were built to estimate the separate associations between cardiorespiratory fitness and physical workload and all cause DP, musculoskeletal and cardiovascular specific DP during the follow-up period (2006–2020). Follow-up time was counted until a DP occurred, the end of the follow-up period, death, migration from Sweden, or turning 65, whichever came first.

To examine the impact of different covariates, four models were initially analysed. Model 1 presents the unadjusted estimates. Model 2 is adjusted for birth year, childhood SEP, cognitive ability and BMI. Model 3 is further adjusted for previous unemployment and model 4 is further adjusted for education. All analyses were adjusted for age. The covariates were chosen because they have been identified as potential confounders in previous studies (Gravseth et al. 2007; Johansson et al. 2012; Karlsson et al. 2008; Karnehed et al. 2007; Krokstad et al. 2002; Sörberg et al. 2013), and may be correlated with choice of occupation and cardiorespiratory fitness as well as being risk factors for DP. All four models are presented in Table 3, whereas in Table 4, model 1 (crude) and one additional model (fully adjusted with all included covariates) are used.

Additional models were built to estimate associations between combinations of physical workload and cardiorespiratory fitness, and the different outcome measures. Potential interaction effects between cardiorespiratory fitness and physical workload were tested using the synergy index (SI) (Rothman 1986). The SI measures if the effect of combined exposure to two factors on an outcome exceeds the sum of the effects of each single exposure separately, when those unexposed to both exposures are used as a reference (Andersson et al. 2005). If SI > 1, there is a synergistic interaction. The 95% CI for the SI were calculated according to Andersson et al. (2005).

## Results

During the follow-up (2006–2020), 10 311 new cases of DP were found. Among these, 2036 cases of DP were due to MSD and 1582 cases of DP were due to CVD.

### Distribution of covariates and cardiorespiratory fitness in levels of physical workload

Having a parent being a farmer or manual worker was more prevalent in the group with the highest physical workload and having a parent in a non-manual position was more prevalent in the group with low physical workload (Table 1). Low cognitive ability, being overweight or obese, having a lower educational level and previous unemployment, were also more prevalent in the group with high physical workload, compared to those with lower physical workload. Furthermore, in the group with high physical workload, a higher proportion also had a low level of cardiorespiratory fitness at conscription.

**Table 1:** Distribution of the covariates and cardiorespiratory fitness across levels of physical workload

		Physical workload					
		Low		Medium		High	
		N	%	N	%	N	%
Age	44–49	53,528	55.5	50,155	55.4	53,196	57.6
	50–54	42,990	44.5	40,305	44.6	39,179	42.4
Childhood SEP	Unskilled	20,131	20.9	27,944	30.9	33,580	36.4
	Skilled	17,441	18.1	21,189	23.4	25,430	27.5
	Assistant	14,288	14.8	10,886	12	7334	7.9
	Intermediate	26,758	27.7	16,846	18.6	10,662	11.5
	Higher	10,608	11	4506	5	1607	1.7
	Farmer	3719	3.9	4382	4.8	8453	9.2
	No SEP	3573	3.7	4707	5.2	5309	5.7
Cognitive ability	Low	14,926	15.5	32,570	36	48,819	52.8
	Medium	57,327	59.4	48,766	53.9	39,965	43.3
	High	24,265	25.1	9,124	10.1	3591	3.9
BMI	Underweight	9,125	9.5	8,693	9.6	8,984	9.7
	Normal	81,327	84.3	73,604	81.4	73,562	79.6
	Overweight	5,436	5.6	7067	7.8	8,403	9.1
	Obese	630	0.7	1096	1.2	1426	1.5
Unemployment days	0	87,412	90.6	76,418	84.5	73,220	79.3
	1–365	6892	7.1	10,174	11.2	13,233	14.3
	> 365	2214	2.3	3868	4.3	5,922	6.4
Education^a^	≤ 9	6095	6.3	16,007	17.7	28,180	30.5
	10–11	18,438	19.1	34,822	38.5	51,081	55.3
	12	12,780	13.2	11,707	12.9	7997	8.7
	≤ 13	59,205	61.3	27,924	30.9	5117	5.5
Cardiorespiratory fitness	Low	26,214	27.2	30,309	33.5	35,439	38.4
	Medium	31,477	32.6	30,337	33.5	32,391	35.1
	High	38,827	40.2	29,814	33	24,545	26.6

*Childhood SEP*  childhood socioeconomic position: unskilled = unskilled manual workers, skilled = skilled manual workers, assistant = assistant non-manual employees, intermediate = non-manual employees at intermediate level, higher = non-manual employees at higher level, no SEP = no parental occupation reported

^a^ ≤ 9 = compulsory school, 10–11 = secondary, 12 = upper secondary, ≤ 13 = university

### Association between covariates and DP

Being older, having a lower childhood socioeconomic position, low or medium cognitive ability, low educational level, and previous unemployment were associated with increased risks of DP (Table 2). Apart from BMI and unemployment days, the associations were stronger with DP due to MSD than with any or CVD DP. Having a high BMI was associated with all three DP outcomes and being underweight was weakly associated with all cause DP.

**Table 2:** Associations between each covariate and disability pension (DP)

Covariate		Any DP N = 10,311	Musculoskeletal DP N = 2036	Cardiovascular DP N = 1582
		HR (95% CI)	HR (95% CI)	HR (95% CI)
Age	44–49	1	1	1
	50–54	1.69 (1.62–1.76)	2.38 (2.15–2.63)	1.57 (1.41–1.75)
Childhood SEP	Unskilled	1.76 (1.59–1.95)	3.33 (2.48–4.47)	2.35 (1.76–3.15)
	Skilled	1.64 (1.48–1.82)	2.93 (2.18–3.95)	2.10 (1.56–2.83)
	Assistant	1.37 (1.22–1.53)	1.82 (1.32–2.52)	1.74 (1.27–2.40)
	Intermediate	1.16 (1.04–1.30)	1.78 (1.32–2.42)	1.38 (1.01–1.88)
	Higher	1	1	1
	Farmer	1.27 (1.12–1.45)	2.45 (1.75–3.45)	1.64 (1.15–2.33)
	No SEP	2.13 (1.89–2.41)	4.14 (2.99–5.74)	2.52 (1.79–3.54)
Cognitive ability	Low	2.56 (2.37–2.76)	4.74 (3.84–5.88)	2.62 (2.15–3.18)
	Medium	1.48 (1.37–1.60)	2.21 (1.78–2.75)	1.50 (1.23–1.83)
	High	1	1	1
BMI	Underweight	1.10 (1.03–1.18)	1.05 (0.90–1.22)	0.99 (0.83–1.18)
	Normal	1	1	1
	Overweight	1.49 (1.40–1.59)	1.78 (1.56–2.03)	1.69 (1.45–1.97)
	Obese	2.63 (2.33–2.96)	2.91 (2.26–3.75)	2.86 (2.13–3.84)
Unemployment days	0	1	1	1
	1–365	2.04 (1.94–2.15)	2.30 (2.06–2.56)	1.70 (1.48–1.94)
	> 365	2.78 (2.60–2.97)	2.69 (2.32–3.14)	1.98 (1.63–2.39)
Education^a^	< 9	2.40 (2.27–2.55)	5.42 (4.64–6.33)	2.90 (2.49–3.38)
	10–11	2.05 (1.94–2.16)	4.17 (3.60–4.83)	2.46 (2.14–2.82)
	12	1.45 (1.34–1.56)	2.41 (1.98–2.94)	1.48 (1.20–1.81)
	< 13	1	1	1

*HR* Hazard ratios 95% CI 95% confidence interval ^a^ ≤ 9 = compulsory school, 10–11 = secondary, 12 = upper secondary, ≤ 13 = university

### Associations between physical workload and cardiorespiratory fitness respectively, and DP

In the crude model (model 1), medium and high levels of physical workload, compared to low physical workload, were associated with an increased risk for all-cause DP (HR for high levels 2.32 95% CI 2.20–2.44), MSD DP (HR for high levels 4.33 95% CI 3.78–4.97), and CVD DP (HR for high levels 2.34 95% CI 2.05–2.67) (Table 3). These associations showed a dose–response like pattern. Furthermore, the strongest associations between high physical workload and DP were due to MSD.

**Table 3:** Hazard ratios (HR) and 95% confidence intervals for the association between physical workload, cardiorespiratory fitness and DP (all cause, MSD, and CVD)

All cause DP			Model 1	Model 2	Model 3	Model 4
		N cases (%)	HR (95% CI)	HR (95% CI)	HR (95% CI)	HR (95% CI)
Physical workload	Low	2063 (2.1)	1	1	1	1
	Medium	3699 (4.1)	1.93 (1.83–2.03)	1.66 (1.57–1.76)	1.59 (1.51–1.68)	1.46 (1.39–1.55)
	High	4549 (4.9)	2.32 (2.20–2.44)	1.86 (1.76–1.97)	1.71 (1.62–1.81)	1.48 (1.39–1.57)
	Total	10,311				
Cardiorespiratory fitness	Low	4225 (4.6)	1.75 (1.67–1.84)	1.51 (1.43–1.59)	1.47 (1.40–1.55)	1.38 (1.32–1.46)
	Medium	3542 (3.8)	1.41 (1.34–1.48)	1.28 (1.22–1.35)	1.27 (1.20–1.33)	1.22 (1.15–1.28)
	High	2543 (2.7)	1	1	1	1
	Total	10,311				
MSD DP						
Physical workload	Low	256 (0.3)	1	1	1	1
	Medium	710 (0.8)	2.97 (2.57–3.42)	2.33 (2.01–2.70)	2.23 (1.93–2.58)	1.84 (1.59–2.14)
	High	1070 (1.2)	4.33 (3.78–4.97)	3.04 (2.63–3.51)	2.80 (2.42–3.24)	2.03 (1.75–2.37)
	Total	2,036				
Cardiorespiratory fitness	Low	856 (0.9)	1.98 (1.76–2.21)	1.56 (1.39–1.76)	1.52 (1.35–1.71)	1.35 (1.20–1.52)
	Medium	710 (0.8)	1.55 (1.38–1.74)	1.33 (1.19–1.50)	1.32 (1.17–1.48)	1.22 (1.08–1.37)
	High	470 (0.5)	1	1	1	1
	Total	2036				
CVD DP						
Physical workload	Low	322 (0.3)	1	1	1	1
	Medium	539 (0.6)	1.78 (1.55–2.05)	1.50 (1.30–1.73)	1.46 (1.27–1.69)	1.29 (1.11–1.49)
	High	721 (0.8)	2.34 (2.05–2.67)	1.81 (1.56–2.08)	1.72 (1.49–1.99)	1.37 (1.17–1.59)
	Total	1582				
Cardiorespiratory fitness	Low	676 (0.7)	1.83 (1.61–2.08)	1.66 (1.45–1.89)	1.63 (1.43–1.86)	1.50 (1.31–1.72)
	Medium	535 (0.6)	1.42 (1.24–1.62)	1.32 (1.15–1.50)	1.30 (1.14–1.49)	1.23 (1.08–1.41)
	High	371 (0.4)	1	1	1	1
	Total	1582				

Model 1: adjusted for age

Model 2: adjusted for age, birth year, childhood SEP, cognitive ability, and BMI

Model 3: adjusted for age, birth year, childhood SEP, cognitive ability, BMI, and previous unemployment

Model 4: adjusted for age, birth year, childhood SEP, cognitive ability, BMI, previous unemployment, and education

A medium or low level of cardiorespiratory fitness, compared to high cardiorespiratory fitness showed increased risks for DP in the crude model (model 1). This was true for all cause DP (HR for low level 1.75 95% CI 1.67–1.84), MSD DP (HR for low level 1.98 95% CI 1.76–2.21), and CVD DP (HR for low level 1.83 95% CI 1.61–2.08). These associations also showed a dose–response like pattern.

When adjusting for birth year, childhood SEP, cognitive ability, BMI, previous unemployment and education, all associations between both higher physical workload and lower levels of cardiorespiratory fitness and DP were attenuated but remained statistically significant (fully adjusted model). The dose–response-like pattern was also less clear in the fully adjusted model.

### Combined effects of low cardiorespiratory fitness and high physical workload on DP

In Table 4 we show groups with combined exposures from cardiorespiratory fitness in three levels and physical workload in three levels, with those with highest fitness and lowest level of physical workload used as a reference category. For each level of cardiorespiratory fitness, the risk of DP increased with increased exposure to physical workload. For each level of physical workload, the risk of DP increased with lower level of cardiorespiratory fitness. Those with the combination of the highest physical workload and the lowest level of cardiorespiratory fitness showed the highest risk. This was true for all cause DP (HR 3.39 95% CI 3.10–3.70), MSD DP (HR 6.21 95% CI 4.93–7.83, and CVD DP (HR 3.91 95% CI 3.09–4.95). In the fully adjusted model, all associations for combined exposure and all DP outcomes were attenuated but remained statistically significant. When adding the effects of the single exposures (high physical workload/high fitness and low physical workload/low fitness), an increased SI, indicating an interaction effect, was shown in the crude model for DP from all causes (HR 1.23 95% CI 1.09–1.39), and from MSDs (HR 1.40 95% CI 1.13–1.74). In the adjusted analyses the risk attenuated but showed the same pattern as in the unadjusted analyses. However, the SIs were no longer statistically significantly increased.

**Table 4:** Hazard ratios (HR) and 95% confidence intervals for the association between separate and combined exposure to low cardiorespiratory fitness and high physical workload on DP (all cause, MSD, and CVD)

			Physical workload					
			Model 1			Model 2		
			Low	Med	High	Low	Med	High
All Cause DP	Cardiorespiratory fitness	High	1	1.83 (1.66–2.02)	**2.26 (2.04–2.49)**	1	1.44 (1.30–1.59)	**1.43 (1.29–1.59)**
		Med	1.27 (1.14–1.42)	2.45 (2.23–2.70)	2.99 (2.73–3.27)	1.15 (1.03–1.28)	1.73 (1.57–1.90)	1.79 (1.63–1.98)
		Low	**1.68 (1.51–1.86)**	3.06 (2.80–3.36)	**3.39 (3.10–3.70)**	**1.39 (1.25–1.55)**	2.00 (1.81–2.20)	**1.95 (1.77–2.15)**
	SI				1.23 (1.09–1.39)			1.14 (0.93–1.39)
MSD DP	Cardiorespiratory fitness	High	1	2.67 (2.06–3.46)	**3.90 (3.03–5.01)**	1	1.71 (1.31–2.23)	**1.74 (1.34–2.26)**
		Med	1.13 (0.83–1.55)	3.64 (2.84–4.67)	5.79 (4.58–7.32)	0.93 (0.68–1.27)	1.92 (1.49–2.48)	2.36 (1.84–3.02)
		Low	**1.81 (1.35–2.43)**	4.86 (3.82–6.18)	**6.21 (4.93–7.83)**	**1.29 (0.96–1.74)**	2.28 (1.78–2.94)	**2.39 (1.87–3.07)**
	SI				1.40 (1.13–1.74)			1.28 (0.90–1.83)
CVD DP	Cardiorespiratory fitness	High	1	1.95 (1.49–2.55)	**2.62 (2.02–3.41)**	1	1.46 (1.11–1.91)	**1.51 (1.14–1.99)**
		Med	1.44 (1.09–1.91)	2.53 (1.96–3.26)	3.38 (2.65–4.30)	1.30 (0.98–1.73)	1.70 (1.30–2.20)	1.86 (1.44–2.42)
		Low	**2.10 (1.60–2.75)**	3.22 (2.52–4.12)	**3.91 (3.09–4.95)**	**1.79 (1.36–2.36)**	2.06 (1.59–2.67)	**2.16 (1.67–2.78)**
	SI				1.07 (0.82–1.39)			0.83 (0.56–1.23)

Model 1: adjusted for age

Model 2: adjusted for age, birth year, childhood SEP, cognitive ability, BMI, previous unemployment, and education

SI = Synergy Index comparing low to high cardiorespiratory fitness and physical workload

HR in bold were used to calculate the SI

## Discussion

### Summary of the findings

In this large cohort study of Swedish men, we found that low cardiorespiratory fitness and exposure to high physical workload were associated with DP (due to any cause, MSD and CVD). These increased risks remained but were clearly attenuated after adjustment for pre-labour market factors such as socioeconomic position in childhood, cognitive ability, and achieved education. The combination of low cardiorespiratory fitness and high physical workload showed higher risks for DP (for all cause and MSD) than would be expected when adding the effects of the single exposures. However, when investigating the interaction effect through a SI, an interaction effect was indicated, but was not significant after adjustments.

### Comparison with previous studies

The demonstrated increased risks of DP from exposure to low cardiorespiratory fitness and exposure to high physical workload in this study are in agreement with previous studies (Emberland et al. 2017; Ervasti et al. 2019; Halonen et al. 2020; Karpansalo et al. 2003).

Our study indicated higher risks of DP (all cause and MSD) from combined exposure to low cardiorespiratory fitness and high physical workload. Only one previous study was found investigating the association between combinations of exposure to low cardiorespiratory fitness and high physical workload on DP (Karpansalo et al. 2002). Karpansalo et al. (2002) found that the combination increased the risk of DP among 1700 Finnish men, which is in line with the present study. In their study the exposure measurement was assessed by multiple self-reported questions measured at the same time as cardiorespiratory fitness was assessed, which could potentially introduce a bias, if those with low cardiorespiratory fitness perceive the physical workload as higher compared to those with high cardiorespiratory fitness. Findings from our study build further on these previous results by using a JEM as a somewhat more objective measurement of physical workload, and with the addition of adjusting for several potential confounding factors from early life, showing that these factors played an explanatory role in the associations between combined exposure to low cardiorespiratory fitness and high physical workload and DP.

The strongest combined association in this study was found for DP due to MSD, which also is in line with the results by Karpansalo et al. (2002). Cardiorespiratory fitness is likely related to overall physical capacity such as muscle strength, balance, and motor function. This may explain the stronger association for MSD DP. However, other studies have also shown associations from combined exposure on risks of all-cause and ischemic heart disease mortality (Holtermann et al. 2016, 2010). Like the results from our study, both of these studies also showed that the combined exposure to high physical workload and low cardiorespiratory fitness were associated with higher risks than for those only exposed to one risk factor.

We found indications of an interaction effect between low cardiorespiratory fitness and high physical workload for all cause DP and MSD DP. However, after full adjustment the estimates were no longer statistically significant. When investigating a potential interaction effect, the previous literature shows somewhat conflicting results. For instance, Holtermann et al. (2010) showed a significant interaction with the two exposures and ischemic heart disease mortality, but a non-significant interaction for all-cause mortality. Future studies are needed before conclusions on potential interaction effects can be drawn.

### Strengths and weaknesses

Major strengths of this study are the large study population, a long follow-up period, the use of objective register data on DP, access to a range of potential confounders concerning pre-labour market factors, and the use of a JEM for the exposure measurement of physical workload. The Swedish JEM for physical workload have shown to be able to predict DP (Falkstedt et al. 2021) and MSD (Badarin et al. 2021), and other studies using similar constructed JEMs suggest that the JEMs can be used as a valid instrument for exposure assessment in large-scale epidemiological studies (Hanvold et al. 2019; Rijs et al. 2014; Solovieva et al. 2012). However, some limitations of the JEM are worth noting. The JEM is based on self-reported data, which is seen as less accurate compared to technical measurements for assessment of physical workload. Yet, the JEM is constructed on self-reported data from a different but large sample of individuals than those investigated in this study and, therefore, provide a more independent measurement on the exposure. A further limitation with the JEM is that the estimated mean-value for an occupation is based on an aggregated level and the possible heterogeneity within the occupation is lost, which could lead to a potential non-differential misclassification of the exposure (Obling et al. 2015). Furthermore, with one single baseline exposure measurement, the study does not account for potential exposure changes over time. It could be assumed that people with different health-conditions change from an occupation with high exposure to an occupation with lower exposure, which could potentially lead to an underestimation of the associations between physical workload and DP. However, a former Swedish report shows that the stability of occupation is rather high, motivating an assumption of relatively high stability of exposure to physical workload over time (Östh et al. 2011).

A further strength of this study is the measurement of cardiorespiratory fitness by a maximal bicycle test, instead of using self-reported data on physical fitness. Self-reported data is generally perceived as less accurate due to reporting bias (Schuler and Marzilli 2003), where for example men tend to overestimate physical fitness more than women (Obling et al. 2015). However, one limitation is the lack of repeated measurements of cardiorespiratory fitness, as well as the long time period between measurements and follow-up. There is limited knowledge on how physical capacity in youth predicts future levels of physical capacity. A recent review showed that individuals´ cardiorespiratory fitness, muscular strength and muscular endurance had moderate stability from childhood and/or adolescence to adulthood, independent of the capacity test being used (García-Hermoso et al. 2022). Research also indicates that people with poor fitness in youth rarely improve their fitness during their lives and that poor fitness early in life may constitute a vulnerability factor (Van Oort et al. 2013). We therefore believe that the tests of physical capacity carried out when the men in our study population were 18–20 years old provide central information for our research questions.

A limitation is that we only have data on men, as only men enlisted to military conscription at the time that data was collected. This makes it hard to draw conclusions on potential associations for women. Another factor to consider is, although we selected covariates based on previous studies, we are unable to exclude potential residual confounding, e.g., from lifestyle factors such as smoking and leisure time physical activity. However, including education as a confounder could be considered a crude proxy for lifestyle factors, as these variables are expected to co-vary (Mäki et al. 2014).

### Interpretation of the results

Our findings that low cardiorespiratory fitness in youth as well as later being exposed to high physical workload were associated with DP due to various diagnoses, highlights the importance of both public health and work environment efforts. Also, the results, possibly indicating an interaction effect between low cardiorespiratory fitness and a high physical workload, emphasize the importance of taking proactive actions in these vulnerable groups. For instance, targeting a balance between physical capacity and physical workload at the workplaces could potentially contribute to a long and healthy working life (Ilmarinen 1989; Karlqvist et al. 2003). To increase the capacity of the workers, several studies have shown that physical exercise at the workplace could lead to reduced musculoskeletal pain and sickness-absence, which are pathways to later receiving a DP (Sundstrup et al. 2020). However, recovery time during working hours is also essential for physically demanding jobs (Korshøj et al. 2015). Furthermore, we have recently found that DP could be prevented by changing from a job with heavy physical workload to a job with lower physical workload (Badarin et al. 2022).

The baseline characteristics of the study population indicate a selection into manual jobs for those with lower levels of cardiorespiratory fitness, lower education, and lower cognitive ability, which could explain a part of the excess DP cases among workers with high physical workload also shown in other studies (Falkstedt et al. 2021; Kjellberg et al. 2016). A recent Swedish study also showed that the level of cardiorespiratory fitness is lower in blue-collar occupations compared to white-collar occupations (Väisänen et al. 2021). One might assume that those with the highest physical capacity enter jobs with high physical workload in order to cope with the physical demands that the job requires. However, the opposite seems to be the case. Since low physical capacity correlates with lower cognitive ability (Åberg et al. 2009), it may be that those with lower cardiorespiratory fitness are more likely to end up in physically demanding jobs with lower educational requirements. The fact that those with low cardiorespiratory fitness in youth was more likely to enter an occupation with high physical workload highlights a vulnerable group and enhances the importance of preventative measurements to increase physical activity already early in life, prior to entering the working life.

## Conclusion

The main findings of this study were that both low cardiorespiratory fitness in youth and later exposure to high physical workload were associated with an increased risk of DP, where workers with the combination of both low cardiorespiratory fitness and a high physical workload had the highest risks (all-cause and MSD DP). This highlights the importance of preventive measures targeting both increased cardiorespiratory fitness in youth and during working life as well as reduced physical workload. Future research should focus on developing interventions for such preventive strategies.

## Data Availability

Data may be obtained from a third party and are not publicly available. The data used for this study were obtained from Statistics Sweden (SCB).
